# Barriers and Facilitators to Expanding User-Administered Injectable Contraceptives in the United States

**DOI:** 10.1097/og9.0000000000000141

**Published:** 2026-01-08

**Authors:** Chase Clark, An-Lin Cheng, Laura Creason, Jennifer Karlin

**Affiliations:** School of Medicine, University of California, Davis, Sacramento, California; Department of Biomedical and Health Informatics, School of Medicine, University of Missouri–Kansas City, Kansas City, Missouri; and Department of Family and Community Medicine, University of California, San Francisco, San Francisco, California.

## Abstract

**OBJECTIVE:**

To identify facilitators and barriers to prescribing subcutaneous depot medroxyprogesterone acetate (DMPA) for user administration.

**METHODS:**

This mixed-methods study comprised surveys (N=422) and interviews (N=34) with family planning experts, defined as health care professionals who see at least three contraceptive visits a month, regarding their awareness, knowledge, and practice of prescribing subcutaneous DMPA for user administration.

**RESULTS:**

We found that 74.5% of professionals reported awareness of subcutaneous DMPA for user administration, but only 34.8% actively prescribed it. Professionals who completed a fellowship in reproductive health, specialized in obstetrics and gynecology, saw a higher number of visits for contraception in a setting with a larger number of pregnancy-potential patients, or worked at a clinic with Title X funding, Planned Parenthood, or a freestanding clinic were more likely to be aware of subcutaneous DMPA for user administration. Negative predictors of awareness included internal medicine specialization, working in a pharmacy, and having a fewer number of contraception options available. Significant predictors for prescribing subcutaneous DMPA if aware of the option include being gender diverse or male, having access to subcutaneous DMPA–specific clinical workflows, and having state Medicaid and private insurance coverage for subcutaneous DMPA. Negative predictors for prescribing if aware include living in a state with abortion restrictions.

**CONCLUSION:**

Our data among experts in contraceptive care highlight that awareness is associated by professional- and institutional-level factors and barriers between awareness of subcutaneous DMPA for user administration and provision that are primarily institutional and systemic. Facilitators for awareness and provision of subcutaneous DMPA for self-administration span multiple domains, including professional's perceived patient barriers, professional demographics, institutional challenges, and government policies.

## Abstract 2

Addressing barriers and implementing facilitators across domains can guide efforts to expand access to subcutaneous depot medroxyprogesterone acetate for user-administration and mitigate disparities in contraceptive provision.

Subcutaneous depot medroxyprogesterone acetate (DMPA) is a progestin-only contraceptive approved for professional administration by the U.S. Food and Drug Administration (FDA) in 2004 for pregnancy prevention. Research demonstrates comparable safety and effectiveness for user administration compared with professional administration,^1–8^ prompting organizations like the Centers for Disease Control and Prevention, Office of Population Affairs, and the World Health Organization to recommend offering the user-administered formulation to individuals of reproductive age.^2,3^ U.S.–based studies highlight interest in user administration, improved continuation rates, and feasibility across diverse populations.^9–18^

Despite its patient-centered benefits and potential to advance equitable health care access, subcutaneous DMPA for user administration remains underutilized in the United States. Limited documentation exists on this gap, with prior studies focusing on health care professional surveys during the coronavirus disease 2019 (COVID-19) pandemic and systematic reviews of implementation strategies.^19–21^ To address this knowledge gap, we conducted a survey and interviews with family planning experts to examine professional awareness, clinical practices, and barriers to offering user-administered subcutaneous DMPA. We hypothesized that barriers to self-administered subcutaneous DMPA span four levels previously identified as categories for care implementation barriers: perceived patient preferences, professional-related factors, clinic-level obstacles, and systemic health care challenges.^22^ This study presents findings and proposes strategies to expand access to this contraceptive option in the United States.

## METHODS

This University of California, Davis IRB–approved mixed-methods study recruited family planning experts by means of convenience sampling and snowball recruitment through email listservs through organizations and individuals who had contacts likely to be experts in sexual and reproductive health. A mixed-method survey (Appendix 1, available online at http://links.lww.com/AOG/E482) with multiple choice, Likert-scale, and free-text questions assessed professional awareness, provision, implementation practices, perceptions of patient suitability, and readiness to prescribe subcutaneous DMPA for user administration. Some survey questions had been validated previously, and the entire survey was piloted with 20 people. Eligible participants included nurses, pharmacists, or physicians (with MD and DO degrees) who self-reported at least three contraceptive visits in the previous month. Surveys were distributed online, excluding professionals not meeting inclusion criteria. Respondents were incentivized to complete the survey through raffle entry for a gift card of $250.

The survey opened in May 2022 for 6 months, yielding 344 complete responses. We reviewed demographics and kept the survey open for another month, with targeted recruitment again through listservs from states with limited responses to ensure geographic diversity, resulting in an additional 78 complete surveys. Surveys with less than 75.0% completion (n=146) were excluded.

Among the respondents, 70.1% (n=296 of 422) expressed interest in follow-up surveys and interviews. Participants who had viewed the Clinical Training Center for Sexual and Reproductive Health subcutaneous DMPA toolkit (n=153) were excluded from the follow-up cohort because the study design aimed to expose participants to the toolkit and to reassess readiness for provision (results forthcoming). Sensitivity analyses compared demographics between the 153 excluded and the remaining 143 respondents. Forty participants were selected at random with a number generator. Thirty-four secondary surveys and interviews were completed. We stopped reaching out to participants when theoretical saturation was reached with the interviews. Sensitivity analysis of the 34 participants who were selected for random follow-up compared with the participants not selected for follow-up interview and survey was conducted to show that those who agreed to follow-up did not differ from the overall cohort and did not differ from the group who accepted interviews and had not viewed the toolkit previously. Follow-up surveys and 1-hour interviews (Appendix 2 and 3, respectively, http://links.lww.com/AOG/E482) were conducted between September 20, 2022, and April 19, 2023, and interviewees were compensated for their time with a $100 gift card. Theoretical saturation was approximated after 34 interviews.^23^

Researchers used inductive–deductive framework to analyze the transcripts individually, noted what stood out to them, and then compared the transcripts until common themes were found among researchers and transcripts across patient, professional, institutional, and systemic domains, in line with previous studies on health care implementation barriers.^22^ Discrepancies in coding were adjudicated by consensus (less than 5.0%).^23^ Barriers and facilitators were categorized across domains (patient, professional, institution, and system), in line with previous studies that outline barriers for health care implementation.^22^

Statistical analyses were performed with R 4.2.1, with χ^2^ tests for categorical variables and independent-sample *t* tests for continuous variables. A value of *P*<.05 was considered statistically significant. Descriptive statistics summarized respondent characteristics and practice settings. Categorical variables are presented as frequencies and percentages, and continuous variables are summarized as mean±SD. Significant factors associated with awareness and lack of awareness of subcutaneous DMPA for user administration were identified through bivariate analyses. Factors of interest included professional sex, age, degree, specialty, fellowship training, years practicing, contraceptive visit quantity per month, percent of patient population with pregnancy capacity, institution of practice, Title X funding status, commonness of DMPA among patient population, geographic location, and insurance coverage. We used χ^2^ tests for categorical variables, Fisher exact tests when cell count was less than five, and independent-sample *t* tests for continuous variables. If normality assumption failed for *t* tests, nonparametric tests were applied. Percentages or averages reflect the proportion or means of respondents within each category.

## RESULTS

**Fig. 1.**
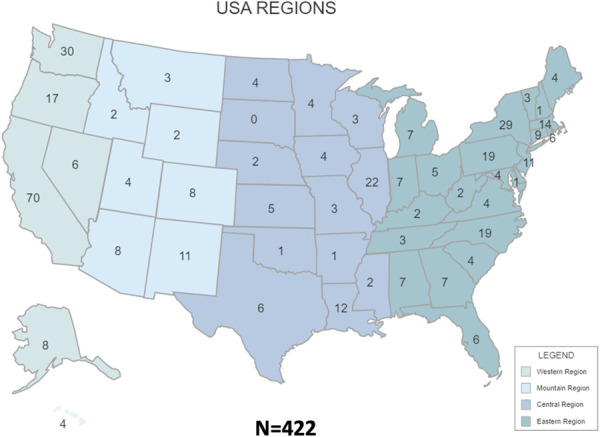
Geographic diversity of participants. Responses were collected from every state in the United States except South Dakota. Only one response was received from Arkansas, Northern Mariana Islands, Delaware, Marshall Islands, New Hampshire, and Oklahoma. The largest number of responses came from California, Washington, New York, Illinois, Pennsylvania, North Carolina, Oregon, Massachusetts, Louisiana, New Jersey, and New Mexico.

Data were collected from a geographically diverse sample of contraceptive experts across the United States and its territories, with responses from all states except South Dakota. Professional demographics and practice characteristics are detailed in Table 1 and Figure 1. Responses to the surveys included 388 that were fully completed and 34 that were 76.0–90.0% completed. Excluded surveys consisted of 16 with 33.0–68.0% completion and 130 with less than 17.0% completion. The response rate was 74.3% conservatively (422 of 568) or 77.1% less conservatively (438 of 568). Sensitivity analysis of survey completion status performed with χ^2^ tests determined no differences in completion rates across age (less than 45 years vs 45 years or more, *P*=.1634), sex (female vs nonfemale, *P*=.2478), or race (White vs non-White, *P*=.5411). This implies survey completion was consistent across demographic groups, supporting the robustness of the results and reducing bias in participation. Similarly, no differences emerged with sensitivity analyses regarding the participants chosen for follow-up compared with the initial cohort.

**Table 1.** Participant Demographics

Gender	
Cisgender female	355 (84.1)
Cisgender male	44 (10.4)
Genderqueer or gender-fluid	10 (2.4)
Transgender male	5 (1.2)
Transgender female	2 (0.5)
None of the above/prefer not to say	6 (1.4)
Age (y)	
Under 35	114 (27.0)
35–44	184 (43.6)
45–54	75 (17.8)
55 and older	49 (11.6)
Degree	
MD/DO	233 (55.2)
NP/PA/DNP*	107 (25.3)
PharmD/BSPharm	34 (8.1)
Masters/PhD	30 (7.1)
RN	18 (4.3)
Practice institution†	
Academic	137 (21.5)
Primary care clinic	113 (17.8)
Planned Parenthood	112 (17.6)
FQHC	64 (10.1)
Pharmacy	47 (7.5)
Other	165 (25.5)
Title X family planning funding	
Yes	166 (39.3)
No	172 (40.8)
Not sure	84 (19.9)
Race and ethnicity‡	
American Indian or Alaska Native	6 (1.3)
Asian	52 (11.6)
Black or African American	36 (8.0)
Hispanic or Latino	25 (5.6)
Native Hawaiian or Pacific Islander	2 (0.4)
None of the above/prefer not to say	14 (3.1)
White	315 (70.0)
Specialty§	
Family practice/family medicine	156 (44.6)
Obstetrics and gynecology	89 (25.4)
Women's health	54 (15.4)
Adult medicine/internal medicine/ID	24 (6.9)
Other	16 (4.6)
Midwifery	8 (2.3)
Pediatrics	3 (0.9)
Fellowship	
No	157 (67.4)
Yes	76 (32.6)
Practice setting‖	
Urban	294 (49.2)
Suburban	141 (23.6)
Telemedicine	82 (13.8)
Rural	77 (12.9)
Frontier	3 (0.5)

FQHC, federally qualified health center; ID, infectious disease.

Values are n (%).

This table shows the demographics of participant health care professionals including identity, education, and workplace institution.

* Fifteen CNM.

† One hundred forty-eight selected more than one institution; 638 total choices.

‡ Twenty-six participants selected multiple choices (total 450).

§ Seventy-two participants did not answer; 350 total choices because of multiple selections.

‖ One hundred twenty participants selected more than one setting; 597 total choices.

*Clark. User-Administered Injectable Contraceptive Use. O&G Open 2025*.

A total of 79.4% of respondents were aware of subcutaneous DMPA for user administration, with 52.0% learning about it during the COVID-19 pandemic (2020–2022). Despite awareness, only 34.8% prescribed subcutaneous DMPA for user administration, and 76.9% of these professionals began prescribing after 2019. This highlights barriers between awareness and provision.

Professional awareness was significantly associated with completing a reproductive health fellowship, obstetrics and gynecology specialization, higher contraceptive visit volumes, urban practice settings, Title X funding, working at Planned Parenthood or freestanding reproductive health clinics, and higher proportions of pregnancy-capable patients. Awareness was inversely associated with internal medicine specialization, pharmacy practice, and having more contraceptive options available (Table 2).

**Table 2.** Factors Associated With Awareness of Subcutaneous Depot Medroxyprogesterone Acetate for User Administration

Factor	Level	Key	Health Care Professionals Who Were Not Aware	Health Care Professionals Who Were Aware	Total Health Care Professionals	*P*
Health care professional completed a reproductive health fellowship	Professional	None	43 (29.5)	103 (70.5)	146 (100.0)	<.001*
		1 y	2 (12.5)	14 (87.5)	16 (100.0)	
		2 y	1 (1.0)	70 (99.0)	71 (100.0)	
Health care professional with obstetrics and gynecology specialty	Professional	Other	61 (23.4)	200 (76.6)	261 (100.0)	.039
		Obstetrics and gynecology	11 (12.4)	78 (87.6)	89 (100.0)	
Internal medicine specialty	Professional	Other	62 (18.8)	267 (81.2)	329 (100.0)	.004
		Internal medicine	10 (47.6)	11 (52.4)	21 (100.0)	
Contraceptive visits per month, median (IQR)	Professional	1=10 or less, 2=11–20, 3=21–30, 4=31–40	2.4 (1.6)	3.5 (2.1)	3.3 (2.0)	<.001
Clinic with Title X funding	Institution	No	65 (26.1)	184 (73.9)	249 (100.0)	.001
		Yes	22 (12.8)	150 (87.2)	172 (100.0)	
Clinic in an urban setting	Institution	No	38 (29.7)	90 (70.3)	128 (100.0)	.004
		Yes	49 (16.7)	245 (83.3)	294 (100.0)	
Clinic is Planned Parenthood	Institution	No	80 (25.8)	230 (74.2)	310 (100.0)	<.001
		Yes	7 (6.3)	105 (93.7)	112 (100.0)	
Clinic is a free-standing reproductive health clinic	Institution	No	84 (18.1)	296 (81.9)	380 (100.0)	.025*
		Yes	3 (7.1)	39 (92.9)	42 (100.0)	
Pharmacy	Institution	No	71 (15.9)	304 (84.1)	375 (100.0)	.026
		Yes	16 (34.0)	31 (66.0)	47 (100.0)	
No. of pregnancy-potential patients, mean±SD	Institution	N/A	55.8±26.7	68.4±26.6	65.8±27.0	<.001
Contraceptive options not offered, mean±SD	Institution	N/A	4.0±2.3	3.1±1.8	3.3±1.9	.001†

IQR, interquartile range; N/A, not applicable.

Values are n (%) unless indicated otherwise.

* *P* value with exact test correction.

† Nonparametric test was performed for this test.

Provision of subcutaneous DMPA for user administration was significantly associated with sex and state political environments. Male and gender-diverse professionals were more likely to prescribe, whereas cisgender female professionals were more likely to be aware but not prescribe (*P*<.001). Professionals in abortion-hostile or middle-ground states, according to Guttmacher Institute's characterization of policy landscapes,^24,25^ were less likely to prescribe if aware of the option (*P*=.05). Institutional workflows and Medicaid/private insurance coverage for subcutaneous DMPA were significant facilitators of prescribing (*P*<.001) (Table 3).

**Table 3.** Factors Associated With Provision if Aware of Subcutaneous Depot Medroxyprogesterone Acetate for User Administration

Factor	Level	Key	Do Not Provide DMPA-SC	Provide DMPA-SC	Total	*P*
Gender, n (%)	Professional	Cisgender female	164 (61.2)	104 (38.8)	268 (100.0)	<.001
		Cisgender male	12 (35.3)	22 (64.7)	34 (100.0)	
		Nonbinary	3 (16.67)	15 (83.3)	18 (100.0)	
Contraceptive options not offered, mean±SD	Institution	N/A	3.5±1.8	2.4±1.2	3.1±1.7	<.001
Workflow for administering DMPA-SC for user administration available in clinic*	Institution	No	171 (66.5)	86 (33.5)	257 (100.0)	<.001
		Yes	8 (12.7)	55 (87.3)	63 (100.0)	
Workflow for administering DMPA-SC available but professional responsible for own education, n (%)*	Institution	No	162 (61.4)	102 (38.6)	264 (100.0)	<.001
		Yes	17 (30.4)	39 (69.6)	56 (100.0)	
Clinic does not have any workflows or has not considered the option, n (%)*	Institution	No	25 (21.0)	94 (79.0)	119 (100.0)	<.001
		Yes	154 (76.6)	47 (23.4)	201 (100.0)	
DMPA-SC covered by Medicaid*	System	No	140 (71.1)	57 (28.9)	197 (100.0)	.039
		Yes	39 (31.7)	84 (68.3)	123 (100.0)	
DMPA-SC covered by private insurance*	System	No	158 (69.0)	71 (31.0)	229 (100.0)	.004
		Yes	21 (23.3)	69 (76.7)	90 (100.0)	
State is considered to have laws protective of reproductive rights according to Guttmacher Institute	System	No	77 (61.6)	48 (38.4)	125 (100.0)	.05
		Yes	91 (50.3)	90 (49.7)	181 (100.0)	

DMPA-SC, subcutaneous depot medroxyprogesterone acetate; N/A, not applicable.

Values are n (%) unless indicated otherwise.

* Asked only for those who are aware of DMPA-SC for user administration.

Professionals aware of subcutaneous DMPA for user administration identified the top barriers to prescribing: lack of patient awareness, limited access to professional education material, and electronic medical record limitations. Professionals prescribing subcutaneous DMPA noted financial barriers and lack of staff support/time for counseling. Professionals predicted 17.3% (CI, 95.0%) of patients not on contraception and 43.7% (CI, 95.0%) of those using intramuscular DMPA would be interested in user administration.

Follow-up interviews revealed perceived patient barriers, including injection schedules and needle fears; professional barriers such as lack of awareness and limited time for injection education; institutional barriers, including lack of standardized workflows; and systemic barriers such as FDA approval status, pharmacy stock issues, and insurance gaps. Facilitators included telehealth visits, mail-order systems, and reminder systems. Illustrative professional quotes regarding barriers and facilitators are listed in Table 4. The most prevalent perceived barriers were at the perceived patient level (n=7) followed by professional level (n=5) with fewer but important institutional (n=2) and systemic (n=3) barriers.

**Table 4.** Quotes From Interviews With Health Care Professionals Supporting Identification of Barriers and Facilitators to Prescribing Subcutaneous Depot Medroxyprogesterone Acetate for User Administration

Level	Barrier	Example Quote	Facilitator	Example Quote
Patient	Patients may forget to inject DMPA-SC	“I really am puzzled about how we would be able to get people to do their every 3 months” “I worry about them remembering it every 3 months” “The hard part is you have to remember to either come in or to inject yourself every 13 weeks or so”	Medication administration reminders	“A lot of times pharmacies … will text you, ‘Oh, it's ready.’ So, things like that: auto refill systems where people get texts or reminders like, ‘Oh, it's time for your Depo. Come pick it up. And this is the window in which you can inject it and still be in a safe contraceptive period’” “I sent them, like, a dosing calendar and some other things, and they said it was really helpful” “I would have a prompt in my EHR that would say, okay, the nurse needs to call so and so. Make sure [they] did [their] Depo”
Patient	Patients may forget how to inject DMPA-SC	“I would be really hesitant that they remember to do everything” “[My] confidence is fairly low that people will remember how to administer in 3 months”	Reminders how to perform injection	“I like the Reproductive Health Access Project handout for user administration. I like that a lot, because it tells you the basics, and then on the back what you're really in for and how you do this to yourself” “The handout on how to administer it would be helpful” “We have handouts on how to keep your needle sterile and how to prepare it and all that kind of stuff”
Patient	Patients may be unaware of DMPA-SC for user administration as a contraceptive option	“I think that's the biggest [barrier]. I think, like, patients not knowing it's an option” “I have not had anybody ask me about it”	Advertise directly to consumers	“When we advertise directly to consumer and then they come in asking for it, so that's an option” “I have not seen any advertising for it, but maybe you should target whatever population you're going for with an ad campaign”
Patient	Patient may not have transportation to their contraceptive appointments	“[Barriers involve] some of the social determinants of health, like living in a rural area or not having transportation” “Sometimes, we have patients, you know, who traveled to us at least for an hour or 2. And so I think for them, that would be, like, a really good choice to kind of offer”	Eliminate transportation needs	“I suppose the self-administered would be a way to facilitate that particular barrier: transportation” “The thing that's keeping people from getting it is not because they have to get an IM shot. It's because they have to go to the actual clinic to have it injected”
Patient	Patients may have a fear of needles or self-injecting	“The patients' biggest barriers, I think needle phobia might be one of them” “People are just afraid of needles. They don't trust needles”	Aid people to overcome their fear of needles	“I think that in the beginning, they would probably be nervous. Like, am I able to do this? Is this going to hurt? Am I going to mess it up? But I think that, you know, eventually, they can totally, like, build the confidence and do it” “I think for most people, it would be totally fine. I mean I have I self-injected other—like, I did a blood thinner for a short time, and I have had that experience. And I think it's much scarier to people than it actually is. And so, you know, we know people give themselves blood thinners, and insulin, and all of these things. And especially if it's only every three months, I don't think that it's nearly as big a deal as people perceive it to be”
Patient	Patients who inject at home may have decreased privacy	“A lot of teenagers like doing things, but if they could do it on their own, too, although … that's something that their parents could find, if they had the needles”	Safely dispose of needles	“I would probably say you can either buy … a sharps container at the pharmacy or, like, order it online, or you can just use like an old laundry detergent container” “I usually tell people to get a glass jar with a metal lid” “We tell our patients to get one of the big laundry—you know, the thick plastic laundry detergent things”
Professional	Professionals lack awareness of DMPA-SC for user administration as a contraceptive option	“I think even at my institution, the majority of the general gynecologists probably aren't aware that it's an option” “I don't think any of my residents or colleagues know about this, sadly” “I wonder if prescriber awareness is a major barrier. And I think, definitely among the nurse practitioners and the PAs that I work with at [redacted], we are not aware that it was an option. Or most of them are not prescribing it or are not aware that they can”	Increase professional awareness	“We all have to get a continuous education somewhere. And so providing that on some big, like, symposiums where they go and target it, I think that would be probably helpful” “More education would be helpful, especially in the primary care setting, just around availability”
Professional	Professionals have ideas of who makes an ideal or unideal candidate for self-administered DMPA-SC	“The ideal patient is young due to delay in fertility, those already injecting hormones, those who want depo but don’t want to come to clinic, self-motivated” “[Nonideal candidates are people who are] they're not good about their own management, mental illness, or just chaotic lives, maybe homeless or no place to keep their stuff”	Eliminate ideas of patient candidacy	“I don't think there's anyone where I would think, oh, they probably can't do this or wouldn't be interested. I think everyone is probably capable or at least should be given the option”
Professional	Professional bias against DMPA	“I think so many people probably undercounsel about depo in general or perceive that patients don't want depo” “I think I probably have my own biases against depo a little bit even though I think it's great for some of my patients”		
Professional	Professional lacks time to teach proper and safe self-injection technique	“I also think that the time it takes to teach a patient is not insignificant” “We don't have sort of a dedicated nurse educator … to do it. It would have to be something that probably I as the provider would do, and it would take up extra clinical time”	Delegate time it takes to teach self-injection to supporting staff	“I have a nurse in my office who then swoops in, does the education” “Some of our pharmacists will teach people”
Professional	Professionals may require a pregnancy test before initiation of user-administered DMPA-SC	“I would say you need to do a pregnancy test before you get the second [DMPA injection]” “I prefer formal [pregnancy] testing. I don't trust anyone”	Do not require a pregnancy test	“I don't think [a pregnancy test is] as important as we previously thought it would be. I think, if patients are educated on why we do it and, again, what the risks are, that this is their life and they're going to be taking charge of their own health, and they can check themselves at home for a pregnancy test” “I think that people can take pregnancy tests at home if they are concerned about potential pregnancy”
Institution	Institution may lack workflows for prescribing DMPA-SC for user administration	“Gosh, I mean, the provider level, just knowledge that it is an option, knowledge of how to bill effectively, and how to accomplish the prior authorizations effectively” “I think I would have to come up with a better workflow if I were doing it over and over every day. Because it is an extra step”	Implementation of protocols or workflows	“Any leg work … that makes it easier for me to know that, like, if I actually prescribe it, it's going to be, like, available for the patient” “I always worry with new things, too, I worry about how to write it. Like, how do I write so that the pharmacy likes it and dispenses it properly? Do I put 4 refills or 5? Yeah. How do I write it to minimize calls from the pharmacy, that I did something wrong?”
Institution	DMPA-SC for user administration may cost the institution	“I feel like some providers may be worried about missed billing opportunities. Like, maybe helping folks understand that admin would probably be on board, because there's not less provider visits, because when they come for their reinjections of IM it's not a provider doing it, it's an MA or a nurse. Which could get could be billed for but frequently isn't” “[The supervisor] was worried that the visit and the money that we're getting from the visits when the patient come every 3 months … that we're going to lose that income”		
Systemic	The FDA has not approved DMPA-SC for user administration	“I mean I feel like one of the things that kind of maybe makes people hesitate or not realize that it's a viable option is the fact that it's approved for provider administration. I know people prescribe and use things off-label all the time. I just feel like, for some reason, contraception seems to be viewed a little bit differently and like, ‘Oh. If the label says this is what it's approved for and how it should be used, that's what we have to do’”	FDA approval of DMPA-SC for user administration	“I truly believe that, in 2004, when the FDA approved it, if they had approved it for user administration and home use, that we would be in a completely different place now” “I feel like, if it had been FDA approved that way 18 years ago, we would've been in a much different situation right now”
Systemic	Pharmacy stock of DMPA-SC is sparse to nonexistent	“We have an affiliated pharmacy, and I don't know that they keep it in stock” “It also isn't available in all pharmacies”	Increase pharmacy stock	“It would make it easier if pharmacies actually stocked it and you know that, when you show up at the pharmacy to get it, that you don't have to go back and get it another day because they don't have it”
Systemic	Insurance coverage	“It's not always covered by insurance and that's a fight” “I mean, I think the biggest [barrier] would be lack of insurance coverage” “I don't have a lot of patients on depo, but I had one patient where it would've been really great, but we couldn't get it covered” “Well, I think the fact that it's not covered in all states makes it something that's less on people's radar” “From an affordability standpoint, if someone were to pay cash for their contraception, it ranges anywhere from like a pack of pills can be less than 10 bucks … I think the most recent price I saw on the depo was somewhere in the $50 to $60 range per injection. That would be for the cash out-of-pocket price for someone”	Insurance coverage of DMPA-SC for user administration	“I'm very lucky, my patients have Medicaid, and Medicaid in [redacted state] pays for it. But I know that it is not on formulary for some of the other insurances”

DMPA-SC, subcutaneous depot medroxyprogesterone acetate; EHR, electronic health record; IM, intramuscular; PA, physician assistant; FDA, U.S. Food and Drug Administration.

Professionals identified several perceived patient-level barriers to user administration of subcutaneous DMPA. Although 85.8% of professionals believed subcutaneous DMPA for user administration was suitable for their patients, fewer than half prescribed it. Interviews highlighted professional assumptions about patient suitability such as patient fear of needles, with biases influencing counseling practices. Facilitators addressing memory challenges included reminder systems, although only 31.5% of prescribing professionals used them. Professionals suggested educational resources detailing safe injection techniques and schedules such as online materials or pamphlets to improve patient confidence.

All interviewees noted lack of awareness among general health care professionals as a significant barrier to expanding the prescribing of subcutaneous DMPA for user administration. Professionals emphasized the need for education through workshops, continuing education courses, publications, newsletters, and patient outreach. Only 39.7% of prescribing professionals discussed subcutaneous DMPA at every contraceptive visit, and 34.2% limited discussions to patients already using DMPA or seeking progestin-only methods. Professional biases further restricted counseling about this option.

Institutional barriers included the absence of standardized workflows for prescribing subcutaneous DMPA for user administration. Structured workflows emerged as a significant facilitator. Professionals also highlighted the time needed to teach self-injection techniques and suggested delegating injection education to nurses or support staff. Pregnancy testing requirements varied, with 55.0% of professionals allowing patients to self-report test results, 15.2% preferring to personally verify a negative pregnancy test, and 18.7% requiring institutional verification.

Telehealth visits were identified as key facilitators, with 81.8% of professionals offering them to address patient barriers. However, only 19.0% of prescribing professionals conducted synchronous telehealth visits, and 24.6% offered asynchronous visits for injection counseling. Most professionals preferred in-person visits for the first injection, with 33.7% observing the patient's first injection and 22.7% administering it directly. Professionals also noted mail-order systems as facilitators, although only 8.4% of Title X clinics and 4.3% of non–Title X clinics offered this option.

State- and government-level barriers included the FDA not approving subcutaneous DMPA for user administration but only for professional administration, pharmacy stock limitations, and economic barriers, including lack of insurance coverage. Professionals expressed greater readiness to prescribe when insurance covered the cost. Systemic facilitators included expanded insurance coverage, which increased professional confidence in counseling patients.

## DISCUSSION

This national study of contraceptive experts highlights a significant gap between awareness and provision of subcutaneous DMPA for user administration. Although 79.4% of contraceptive experts are aware of this option, fewer than half actively prescribe it, consistent with findings from prior studies.^18^ General practitioner awareness is likely even lower, emphasizing the importance of targeted education for nonspecialists.

Expanding access to subcutaneous DMPA requires addressing barriers between awareness and provision. Sex differences in provision may reflect the limited number of male and gender-diverse contraceptive experts in the field and the likelihood that they are more inclined to seek out and advocate for alternative contraceptive options that support patient autonomy than the majority of professionals. State political environments also influence provision, underscoring the importance of policy supporting contraceptive access, particularly in abortion-hostile states.^26–29^

Professional-perceived patient-level barriers such as self-injection fears often stem from professional biases rather than actual patient limitations; previous studies indicate that needle fears are contextual and decrease over duration of use.^10,13^ Needle fears can be mitigated through guided instruction during the first injection and educational materials.^10,21^ Professional bias in assessing patient suitability influences counseling practices and perpetuates limited patient awareness of this option because professionals are a key conduit to information about contraceptive options.^30,31^ Expanding access to subcutaneous DMPA is particularly important for minoritized populations, who often prefer autonomous contraceptive methods; overcoming professional misconceptions is critical for equitable access.^18,32,33^ A prior study found only 1 in 488 professionals used DMPA themselves compared with 41.7% who used long-acting reversible contraception, suggesting that low personal use of DMPA among health care professionals may contribute to its underutilization.^34^ Concerns about pregnancy testing before initiation can be addressed by recommending self-monitoring for pregnancy symptoms or home testing.

Institutional barriers such as the absence of standardized workflows complicate implementation. Toolkits addressing workflows, billing, advocacy, patient education, and counseling can help overcome these barriers. A toolkit designed to assist professionals with workflows, billing, advocacy, patient education, and counseling was identified to address these institutional barriers.^35^

Finally, FDA approval for subcutaneous DMPA for self-administration is a key policy and system-level barrier. Approval would increase professional confidence and expand insurance coverage, reducing economic barriers. Increased demand through expanded prescribing could also improve pharmacy stock availability, further enhancing access.

This is the only national survey of professional awareness and provision of subcutaneous DMPA for user administration in the United States. Strengths of this study include assessment of populations both aware and unaware and both actively prescribing and not prescribing subcutaneous DMPA for user administration, allowing assessment of barriers between awareness and provision. Because experts in contraception were targeted, this large sample of professionals who are aware of subcutaneous DMPA option could assess for trends in knowledge, provision, and practices, as well as barriers and facilitators to provision. Generalizability, however, is limited given its inclusion of only contraception experts, and although the geographic representation in this study was widespread, limitations with missing data from certain U.S. states and territories remained.

Expanding access to subcutaneous DMPA for user administration requires reducing professional biases through education initiatives, developing standardized workflows, and increasing insurance coverage. Further research demonstrating improved continuation rates and long-term viability may support FDA approval, opening additional opportunities for access. Addressing barriers across all levels will empower patients with greater control over their reproductive health.
